# High Accuracy Passive Magnetic Field-Based Localization for Feedback Control Using Principal Component Analysis

**DOI:** 10.3390/s16081280

**Published:** 2016-08-12

**Authors:** Shaohui Foong, Zhenglong Sun

**Affiliations:** 1 Engineering Product Development (EPD) Pillar, Singapore University of Technology & Design (SUTD), Singapore 487372, Singapore; 2 International Design Centre (IDC), Singapore University of Technology & Design (SUTD), Singapore 487372, Singapore; zhenglong_sun@sutd.edu.sg

**Keywords:** artificial neural networks, magnetic sensors, principal component analysis, signal mapping, linear actuators

## Abstract

In this paper, a novel magnetic field-based sensing system employing statistically optimized concurrent multiple sensor outputs for precise field-position association and localization is presented. This method capitalizes on the independence between simultaneous spatial field measurements at multiple locations to induce unique correspondences between field and position. This single-source-multi-sensor configuration is able to achieve accurate and precise localization and tracking of translational motion without contact over large travel distances for feedback control. Principal component analysis (PCA) is used as a pseudo-linear filter to optimally reduce the dimensions of the multi-sensor output space for computationally efficient field-position mapping with artificial neural networks (ANNs). Numerical simulations are employed to investigate the effects of geometric parameters and Gaussian noise corruption on PCA assisted ANN mapping performance. Using a 9-sensor network, the sensing accuracy and closed-loop tracking performance of the proposed optimal field-based sensing system is experimentally evaluated on a linear actuator with a significantly more expensive optical encoder as a comparison.

## 1. Introduction

The measurement of position is paramount for control of devices in a multitude of medical, automation, manufacturing and industrial environments. Of interest is the precise control of translational motion in linear actuators, such as voice coil motors (VCMs) and ironless motors, over large (with respect to resolution) travel distances. Here a distributed sensing approach harnessing a spatial network of magnetic sensors is used concomitantly with trained artificial neural networks (ANNs) to provide accurate real-time position information for subsequent control. To optimally reduce the number of required inputs during ANN mapping, the statistical technique of principal component analysis (PCA) is innovatively applied as a pseudo-filter for the ANN to improve computational efficiency while retaining high accuracy.

A major advantage of field-based sensing systems is their ability to function in harsh conditions. Magnetic fields are invariant to temperature, pressure, radiation and other environmental factors. Capitalizing magnetic fields for orientation/position sensing is not new as evident by Raab’s et al. [1] magnetic tracking system which was introduced more than three decades ago. Compared to other non-contact sensing systems such as optical [2] and vision sensors [3], magnetic sensors do not require ‘a line of sight’, permitting sensing across multiple non-ferromagnetic mediums. Despite major advancement in miniaturization and magnetic sensing technology where modern sensors possess small physical footprints and high sensitivity and bandwidth [4,5], the deployment of magnetic sensors for feedback control is under exploited and can be extremely promising as shown by recent applications in localization of medical instruments [6,7].

Magnetic localization can be categorized into two approaches that involve harnessing active or passive fields. Localization utilizing artificially generated electromagnetic fields can be either pulsed or static and possess superior measurement ranges and better immunity to background geomagnetic noise. However, active fields require power, which can be provided internally using a battery or externally via constricting tethering wires which are less desirable for applications such as medical devices [6,7] that require compact and lightweight footprints. Permanent magnets provide static magnetic fields with zero power and can be embedded directly into the target for non-obtrusive localization and tracking. Both approaches, however, like all sensing principles and systems, require a correspondence between the measured field and instantaneous position/orientation.

The main difficulties in cultivating position-field correspondence are the complexities of analytical field models and absence of bijectivity (both injective and surjective or encompassing one-to-one and onto correspondence) between field measurements and position/orientation. In a non-bijective relationship, multiple positions/orientations share a common field measurement value. It is clear that without bijection, associating an arbitrary field measurement with a unique position is difficult. While researchers have attempted to isolate the linear field region (where the magnetic flux density with respect to distance can be approximated by linear relationship) with sensor arrays [8] and directly overcome the non-linearity via a compensating coil [9] for unique sensing as well as utilize principle of phase commutation [10], a more robust approach for compensating and characterizing the non-linearity as introduced in [11] is adopted here, which offers greater potential for high precision localization.

Another critical issue is the manner and speed of extracting position from field measurements to satisfy the stringent requirements of feedback control. While theoretical field models for the prediction of fields in space are available, they are often highly complex and not in a tractable form for direct inverse computation operations, requiring computationally heavy non-linear optimization methods [12,13,14] which are unsuitable for real-time operations. As illustrated in [15,16], ANNs provide a model free method to map concurrently field measurements to positions with high degree of accuracy where each input of the ANN is linked with a dedicated sensor output. Moreover, ANNs are computationally efficient during online operation and utilized as real-time robust controllers [17], parameter estimators [18] and state observers [19] of motors. A potential issue with harnessing numerous concurrent measurements is the complexity and computation requirements associated with an ANN containing an excessive number sensor inputs.

In image processing, PCA has been diligently adapted to condense a set of highly correlated images to be approximately represented by a significantly smaller set of eigenimages [20] as well as an essential element of an algorithm to induce greater color contrast in RGB images [21]. As magnetic field measurements are highly correlated due to spatial proximity between sensors, the measurements by a large sensor network can be optimally represented by a smaller set of eigenspaces. Within these transformed spaces, obtained from the linear combination of the original sensing axes, only the major principal components are required for ANN mapping. The remainder of this paper presents the following:

- A method using concurrent field measurements of a moving permanent magnet (PM) to infer its precise position in real-time is presented. This method employs a sensor array to provide bijective relationships between measurements of magnetic flux density (MFD) and position as well as spatially extending the sensing range. Instead of directly mapping sensor outputs to position, PCA is used to determine an optimized set of transformed measurements for ANN mapping.
- Through numerical simulations, the effects of geometric parameters (including the PM dimensions and sensor spacing and location) on sensing accuracy is examined. Simulated measurements are corrupted with artificial Gaussian noise to explore practical implementation issues of the system.
- Using an ironless brushless linear motor as a platform for analysis, the experimental performance of a 9-sensor field-based system using PCA optimized ANN mapping is investigated. The tracking error resulting from the closed-loop control of the system using the field-based system is compared with an optical encoder. The tracking performance using the field-based system gives a similar response of that obtained using the optical encoder with 1 µm resolution.

## 2. Materials and Methods

To harness magnetic fields for absolute precision sensing in translational motion, a stationary sensing system is designed to determine motion of the moving magnetic source with the following considerations:

1. A distributed spatial network of sensors to uniquely relate position of a magnetic source to its measured field from the sensors, and
2. An approach using ANNs and PCA to optimally relate multiple concurrent field measurements to position coordinates and minimize time-consuming computation.

**Figure 1:**
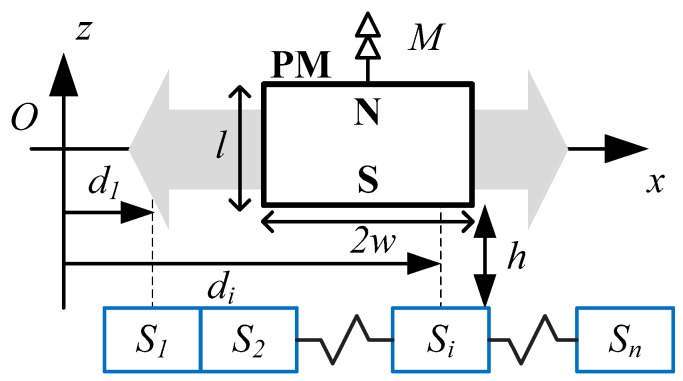
Schematic of a moving PM source and stationary sensors sensing configuration.

For applications of PM-based devices undertaken here, time-invariant PM sources are considered since the effects of electromagnets (with known currents) can be actively compensated or accounted for by modeling them as equivalent magnets [22]. Consider the single-source-multi-sensor configuration shown in Figure 1, where a lateral array of fixed sensors is placed at an offset of *h* from the PM source of a rectangular cuboid geometry (length *l*, width 2*w* and thickness *c*) undergoing horizontal motion (along *x*-axis). The magnetization axis **M** = *M***e_z_** of the PM is perpendicular to the motion path *x*. The aggregate MFD as measured by a network of *n* single-axis sensors at an arbitrary position of the magnetic source can be denoted by Equation (1):

(1) $$Β\left(x,h\right)={\left[\begin{array}{ccccc} f_{1}\left(x,h\right) & \cdots & f_{i}\left(x,h\right) & \cdots & f_{n}\left(x,h\right) \end{array}\right]}^{T}$$

where *f_i_* is the individually measured MFD along the *z*-axis (**e_z_**) from the *i*th sensor at the absolute lateral position *x* of the magnetic source. The vector **B** contains the individual *f_i_* for each sensor, which could represent analytical field models (single dipole [23], distributed multipole model [24,25] or hybrid [26]) or experimental field measurements at each spatial location *d*_i_.

Field-based position sensors require an inverse model that solves for the position x from measured **B**. Essentially, the inverse expression of **B**^−1^, is necessary and sufficient for field-based sensing. However, extracting an analytical expression for *f_i_*^−1^ is difficult due to the high degree of non-linearity and non-uniqueness of analytical field models [27,28]. A numerical method capable of handling discontinuities at the magnet surface involving manipulation of the scalar magnetic potential to compute magnetic fields in a current free space around a magnet can be found in [29].

### 2.1. Inducing Bijectivity with a Spatial Network

Due to symmetry, the measured MFD is spatially symmetric about the centerline of the *i*th sensor:

(2) $$f_{i}\left(x-d_{i},h\right)=f_{i}\left(-(x-d_{i}),h\right)$$

In addition, as the field radiating from a PM is finite, the sensor is unable to detect the change in magnetic field when the PM position exceeds a certain threshold:

(3) $$\left|f_{i}(x-d_{i},h)\right|\{\begin{array}{c} >0\text{ for }\left|x-d_{i}\right|\leq R(h)\\ =0\text{ otherwise} \end{array}$$

where *R* is a specified threshold dependent on the separation distance *h*. While the sensor has a lateral sensing range of 2*R* or *d_i_* ± *R* in Equation (3), its effective positional sensing range is only *R*, [*d_i_* − *R*, *d_i_*] or [*d_i_*, *d_i_* + *R*], because of its inability to distinguish between the two symmetric magnetic fields in Equation (2). The issue with symmetry is that multiple *x* locations result in the same *f_i_*(*x*) value, preventing these locations from being distinguished and hence unique from one another using only *f_i_*(*x*). In other words, for a given continuous interval *x*, *f_i_* must be a strictly increasing or decreasing monotonic function if the entire interval were to be unique. This criterion can be assessed by analyzing the spatial derivative of *f_i_* $\left(\frac{\partial f}{\partial x}\right)$.

**Figure 2:**
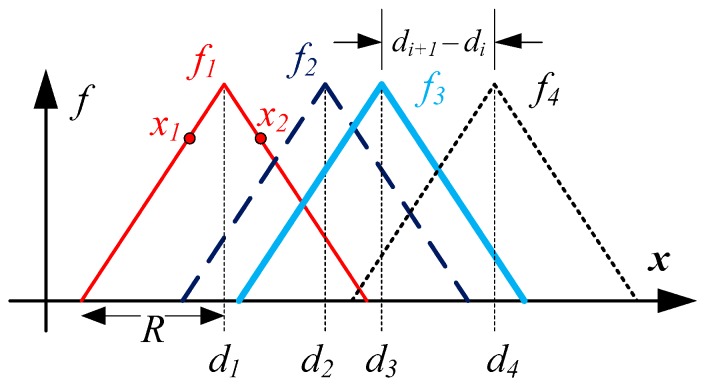
Illustration of concurrent field measurements at various spatial locations.

However, concurrent measurements **B** of multiple sensors do not exhibit the above symmetry because each *f_i_* has distinct axis of symmetry about *d_i_*. As best illustrated using Figure 2 where *f_i_* is approximated by a triangle function for simplicity, the composite vector **B**(*x*) will be unique for ‖B(x)‖>0 as long as *d_i_*_+1_ – *d_i_* < *R* (*i* = 1,…,*n*–1). This stipulation ensures that local axes of symmetries inherent due to Equation (2) are no longer present. As illustrated in Figure 2, while *x*_1_ and *x*_2_ may share the same *f*_1_ value, *f*_1_(*x*_1_) = *f*_1_(*x*_2_), the composite **B** at both values are different and hence unique, $Β\left(x_{1}\right)\neq Β\left(x_{2}\right)$. Moreover, the effective positional sensing range of the sensor network is extended to [*d*_1_ − *R*, *d*_n_ + *R*] as long as ‖B(x)‖>0 throughout that specified range for *x*. In summary, the spatial network of sensors induces uniqueness for **B** as well as increases the range where this uniqueness holds.

Mathematically, if the forward model **B** is bijective, the inverse model **B**^−1^ exists and is bijective as well. This property allows measurements to be mapped uniquely to position coordinates, which is the fundamental mechanics of a sensing system. As analytical solutions to the inverse model are not available (especially if numerous sensors are involved), a function fitting approach is adopted to solve for **B**^−1^ for real-time feedback. With this mapping approach, the desired inverse model **B**^−1^ is approximated by a fitted analytical artificial function. Look-up tables (LUT) and conventional least squares (LS) using basis functions of polynomials and sinusoidals are commonly used methods in creating such mappings but ANN mapping is preferred as the latter are more adaptable and scalable when managing multiple inputs and outputs.

### 2.2. PCA Optimized ANN Mapping

**Figure 3:**
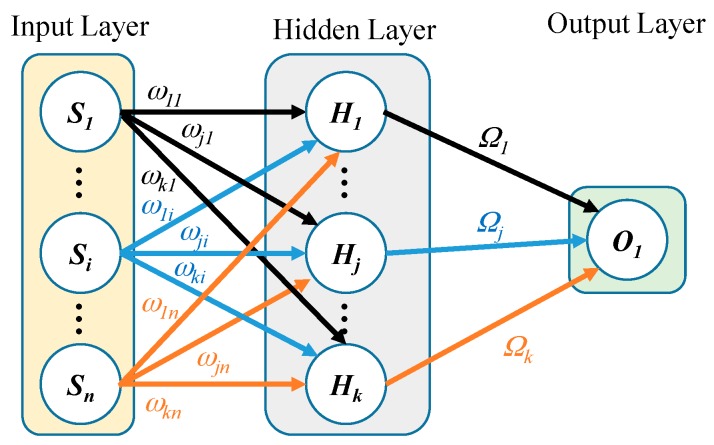
Two layer fully-connected feedforward ANN used for functional mapping. (Biases not shown).

Feedforward ANNs can be trained with backpropagation supervised learning to fit a desired set of inputs to a corresponding set of outputs by iteratively adjusting the weighting coefficients and biases in the network to minimize the root mean-squared error over all data pairs. For this application (estimation of *x*), a fully-connected two layer (single hidden layer) neural network architecture is used as shown in Figure 3. This network consists of *n* nodes in the input layer (field measurements by all sensors **B**), *k* neurons in the hidden layer and a single output neuron (*x* position at ***B***). In order to construct this mapping, the bijective range of motion [*d*_1_ − *R*, *d*_n_ + *R*] is discretized into *N* data points, resulting to total of *N* ANN training-target sets. The positional estimate ${\hat{x}}_{v}$ of the neural network can be mathematically represented as:

(4) $${\hat{x}}_{v}=\Omega \left[\sigma \left(\text{ω}B_{v}\right)+b\right]+c$$

where σ is the sigmoid activation function: *σ*(*θ*) = 1/[1 + exp(–*θ*)], **ω** is a *k* × *n* matrix containing the weighing coefficients ω*_ji_* between the *i*-th input node and the *j*-th hidden node, **Ω** is a 1 × *k* matrix containing weight coefficients **Ω***_j_* between the *j*-th hidden node and output node, **b** is a vector containing the biases of each of the *k* hidden nodes, *c* is the bias of the solitary output node, and the subscript *v* (1≤v≤Ndata points) is an integer representing the training set index. For this single hidden layer network, there are a total of (*n* + 1)*k* weighing coefficients (**Ω**, **ω**) and (*k* + 1) biases (**b**, *c*) which are determined offline during backpropagation training. To numerically execute Equation (4) for every estimate, it will consist of the following simple and efficient scalar arithmetic operations: (*n* + 1)*k* multiplications, (*n* + 1)*k* additions, and *k* Sigmoid functional evaluations. The root mean squared error (RMSE) defined in Equation (5) is used to evaluate of the performance of the neural network:

(5) $$\text{RMSE}=\sqrt{\frac{1}{N}\sum_{v=1}^{N}{\left(x_{v}-{\hat{x}}_{v}\right)}^{2}}$$

**Figure 4:**
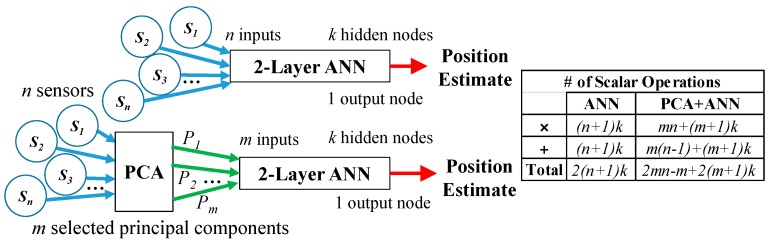
Using PCA to optimally reduce the input dimension for ANN mapping.

Due to spatial proximity of sensors and PM, the field measurements are correlated. Through the orthogonal linear transformation of the PCA, the individual measurements by the sensors (*S*_1_, …, *S_i_* …, *S*_n_) can be represented by the principal components (*P*_1_, …, *P_q_*, …, *P_n_*) such that the greatest variance of the measurements lie on the 1st principal component *P*_1_, the second greatest on the 2nd principal component *P*_2_ and so on [30]. Rather than using correlated sensor field measurements as individual inputs to the ANN and graphically illustrated in Figure 4, an optimized subset of *m* principal components (from the full set of *n* principal components) can be applied to Equation (4) directly and this represents an advantage of requiring fewer ANN inputs while retaining the critical bijectivity of **B**.

Collating individual field measurements into the matrix **A** = [**B**_1_ … **B***_v_* … **B***_N_*], the full suite of *n* principal components of **A** can be determined by finding an orthonormal matrix **P** such that the covariance matrix of **G** (that contains the collated transformed field measurements of **A**) is a diagonal matrix [30]:

(6) $$C_{G}=GG^{T}/n$$

where **G** = **PA** and **P** is a square *n* × *n* matrix obtained from the offline computation of the singular value decomposition (SVD) or eigenvectors of:

(7) $$C_{A}=AA^{T}/n$$

The rows of **P** contain the *n* principal components coefficients of **A**; and the eigenvector with the highest associated eigenvalue is the (first) principal component. The relative numerical value of the eigenvalues denotes the statistical significance of each principal component. To explicitly compute the *q*th principal component from *n* sensor measurements, the following arithmetic expression is used:

(8) $$P_{q}=C_{q}B$$

where **C***_q_* is a 1 × *n* vector constructed from the *q*th row of **P**. Numerically computing *m* principal components requires *mn* scalar multiplications and *m*(*n* − 1) scalar additions. Note this is independent on the ANN architecture (*k*).

**Figure 5:**
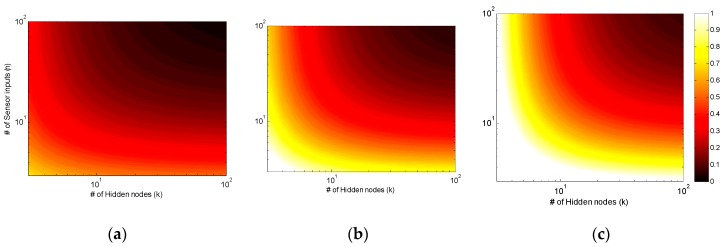
Ratio of number of arithmetic operations using PCA-ANN to ANN-only mapping. Lower ratios (darker areas) denote instances of *k* (# of hidden nodes) and *n* (# of ANN inputs) where PCA-ANN is computationally more efficient. (**a**) *m* = 1, (**b**) *m* = 2, (**c**) *m* = 3.

A detailed mathematical comparison of the number of scalar arithmetic operations required for ANN computation with and without PCA is also illustrated in Figure 4 (sigmoid function evaluations are omitted as they do not change with PCA). As the number of arithmetic operations vary nonlinearly with both *m*, *n* and *k*, the ratio of the total number of arithmetic operations of PCA-ANN to ANN only mapping is computed at various *n* and *k* values and illustrated with a log-scale contour plot in Figure 5 when the first, first and second and first three principal components were used. Hence if the ratio is less than 1, it will represent computational advantage of the PCA-ANN approach over the ANN-only approach.

## 3. Results

### 3.1. Numerical Simulation

This section provides a numerical investigation in the effects of PCA, geometric configuration, sensor spacing and noise corruption on the accuracy of the magnetic field-based sensing. Without loss of generality, a uniformly magnetized cuboid PM (2*w* = *c*, *l*) is used as example for clarity in illustration. The simulations are presented in non-dimensional form to facilitate parametric studies or for design analysis. For this, we normalize lengthwise parameters and variables to *w*, and the magnetic flux density (MFD) in Equation (1) to *μ_o_M_o_* where *μ_o_* is the magnetic permeability of free space; and *M_o_* is a specified residual magnetization (or magnetic moment per unit volume):

(9) $$F=f/M_{o}\mu_{o}\text{, }X=x/w$$

Normalizing the lateral displacement with respect to the PM width creates a useful reference point at unity (when *d*_1_ = 0) where it defines the PM boundary’s projection on *x* regardless of the actual dimensions and parameters of the system. The absolute field sensitivity (AFS) is defined by the partial spatial derivative:

(10) AFS=|∂F/∂X|

#### 3.1.1. Singular Sensor Geometrical Considerations

The effects of three specifications are chosen for evaluating the sensing system, signal-to-noise ratio (SNR), resolution and range. For field-based sensors, SNR corresponds to the relative magnitude of the MFD while AFS impacts the resolution and range. Higher MFD measurements and AFS correlates with superior SNR and smaller resolutions. Increasing the spatial domain where AFS is elevated will extend the sensing range. Hence it will be pertinent to examine the combined spatial effects of the separation ratio *β* = *h*/(2*w*) and geometric aspect ratio *γ* = *l*/(2*w*) of the PM on the simulated field and its corresponding sensitivity.

**Figure 6:**
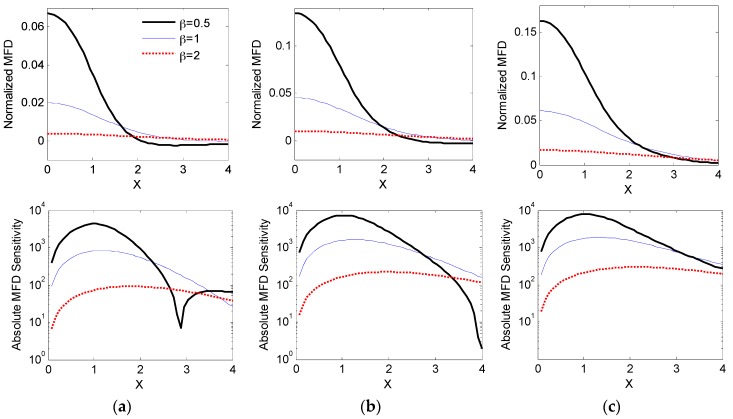
Normalized MFD (top) and absolute sensitivity (bottom) for various aspect and separation ratios; *γ* = *l*/(2*w*) and *β* = *h*/(2*w*). (**a**) *γ* = 0.25, (**b**) *γ* = 1 and (**c**) *γ* = 4.

Using Equations (9) and (10) and employing a spatial resolution of 0.001, the normalized MFD and AFS for a zero-centered (*d*_1_ = 0) single sensor configuration (*S*_1_, *n* = 1) are evaluated for a variety of *β* and *γ* values. The results are compiled in Figure 6, where the top, middle and bottom rows illustrate the numerically simulated MFD and AFS for *γ* = 0.25, 1 and 4 respectively. Within each plot, the effects of *β* (=1, 0.5 and 2) are compared. For all plots, the lateral displacement is spatially zeroed (*d*_1_ = 0) such that the centerlines of the PM and sensor align vertically. In addition, the field sensitivity is presented in logarithmic scale to accentuate the differences and only the positive spatial domain (0≤X≤4) is illustrated as the magnetic field is symmetric about *X* = 0.

#### 3.1.2. Dual-Sensor PCA ANN Mapping Analysis

As a single sensor system is unable to provide a bijective relationship required for a sensing system, the parametric analysis is extended to investigate the relative placement of two sensors on mapping performance. Consider a dual-sensor configuration (*S*_1_,_2_, *n* = 2), where the parameters (*β* = 0.5, *γ* = 0.25) are selected to yield the highest AFS per unit peak MFD.

**Figure 7:**
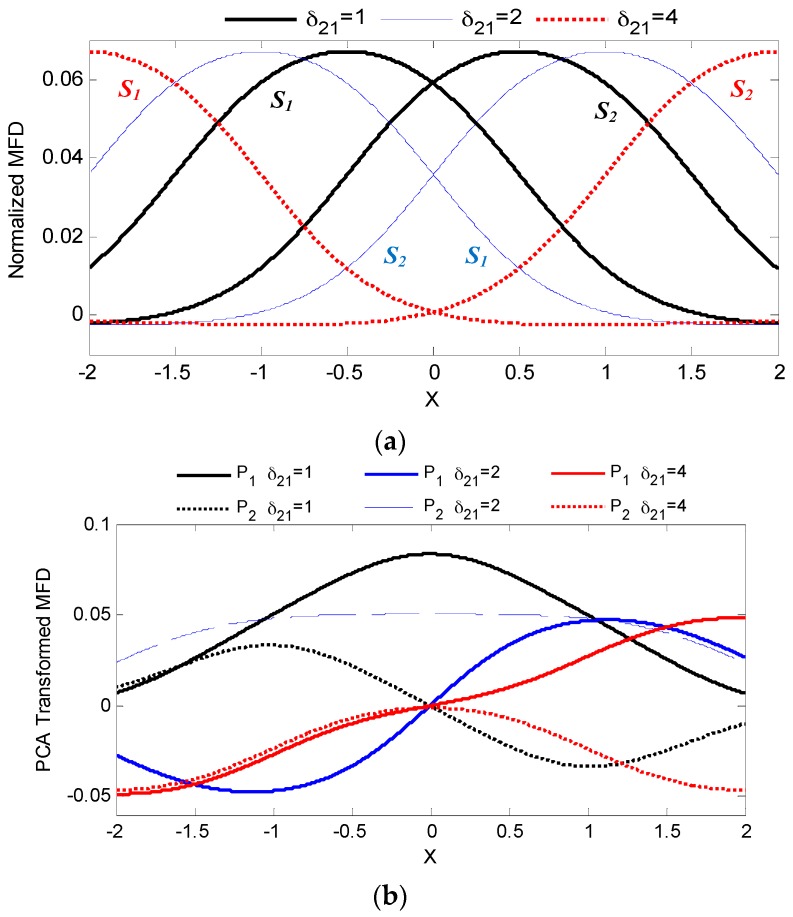
Concurrent MFD measurements (**a**) and transformed principal components (**b**) from three 2-sensor (*S*_1_ and *S*_2_) configuration of varying normalized sensor spacing.

As will be shown, analysis of this dual-sensor configuration provides a fundamental understanding on the design of a more extensive multisensor system. With 0.001 spatial resolution for a total of 4001 data points, Figure 7a visually graphs the simulated effect of the normalized sensor spacing, *δ*_21_ = (*d*_2_ − *d*_1_)/*w*, on the corresponding measured MFD over the domain of −2≤X≤2. Applying PCA using the process outlined in Equations (6) and (7), the two principal components for each of the three distinct normalized sensor spacing (1, 2 and 4) are compared in Figure 7b. For each sensor spacing, the PCA coefficients and associated variability for both principal components are tabulated in Table 1.

**Figure 8:**
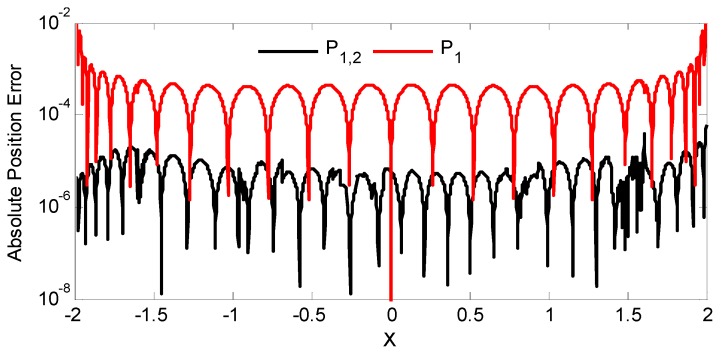
ANN absolute lateral position mapping error at *δ*_21_ = 4 using both and single principal components.

The table also includes the resulting RMSE from utilizing the PCA transformed MFD for ANN mapping. The ANN used for mapping simulation has a single hidden layer architecture with 10 hidden nodes (*q* = 10). Figure 8 spatially illustrates the mapping error resulting from using only the first and both principal components when *δ*_21_ = 4.

#### 3.1.3. Multi-Sensor PCA ANN Mapping Analysis

**Figure 9:**
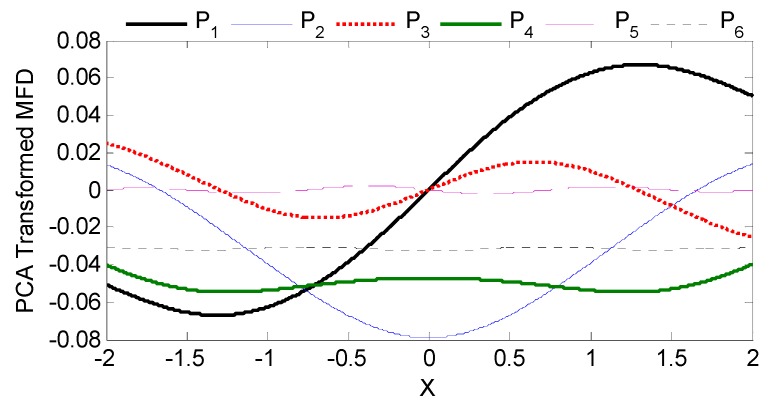
Spatial representation of all six principal components for a 6-sensor system.

The alternative design configuration of three dual sensor pairs depicted in Figure 7a can be perceived as a 6-sensor configuration (*S*_1,2,3,4,5,6_; *n* = 6), with variable spacing. This continued analysis illustrates how effortlessly PCA scales with larger sensor networks while offering the added advantage of improved noise attenuation. The same PCA process as outlined in Equations (6) and (7), can be applied to these six inputs and the six resultant principal components are spatially plotted in Figure 9. The relative variability of each principal component expressed as a percentage and PCA coefficient matrix are consolidated in Table 2.

**Figure 10:**
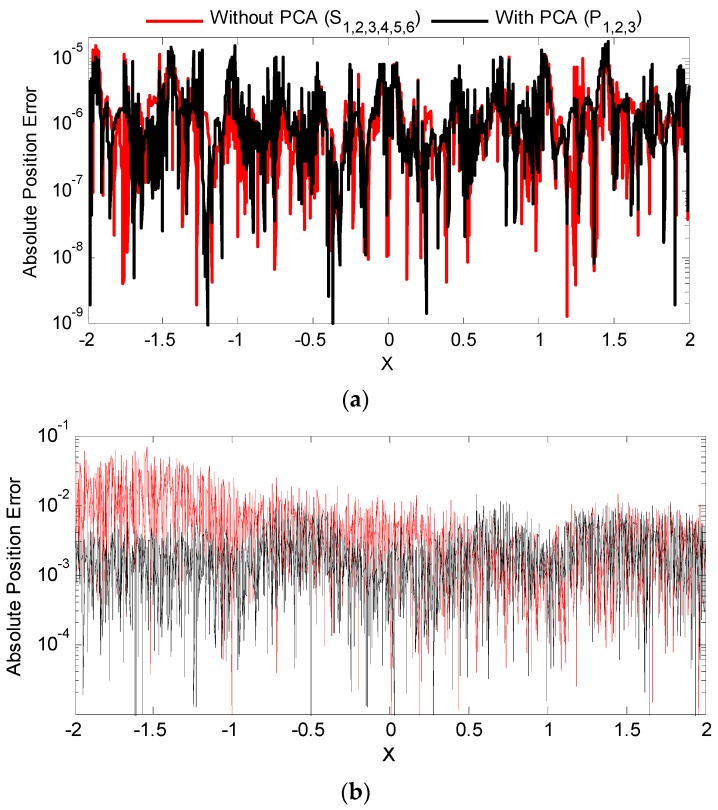
Absolute error comparison between PCA assisted mapping and mapping without PCA at different levels of noise corruption. (**a**) Zero Gaussian noise; (**b**) 1% Gaussian noise.

Employing only the first three principal components, the absolute position error arising from ANN mapping is spatially presented in Figure 10a. The corresponding absolute position error obtained if all six sensor simulated measurements were feed directly into the ANN without PCA transformation was included as a comparison. Finally independent Gaussian noise (with variance equal to 1% of the total variance of a single channel measurement) were simulated in each sensor channel in Figure 7a and Figure 10b illustrates the resultant effect of noise on the absolute position error for the two cases in Figure 10a. For completeness, Table 2 evaluates the effects of various noise levels on the RMSE when the ANN inputs are the raw sensor measurements or processed three main principal components.

### 3.2. Experimental Investigation

**Figure 11:**
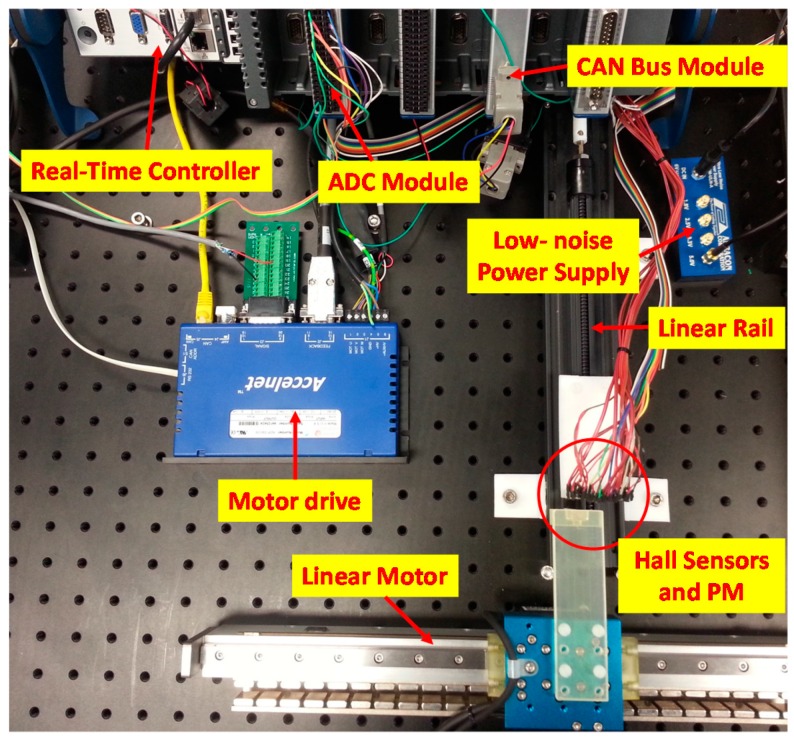
Experimental setup.

Figure 11 shows the experimental set-up that consists of an ironless brushless linear motor driven by a digital servo drive (Accelnet ADP-055-18, Copley Controls, Canton, MA, USA) with optical linear quadrature encoder (RGH41X30D05A, 1 μm resolution, Reinshaw, Gloucestershire, UK), a reconfigurable embedded control and acquisition system (CompactRIO CRIO-9082 populated with a 250 kS/s 16-bit 16-channel ±5 V Analog to Digital Converter (ADC) module NI 9205 and 1 Mb/s 1-port CANopen module NI 9881, National Instruments, Austin, TX, USA) and an ultra low-noise power supply (ABPSM-ULN-A, Abracon, Irvine, CA, USA). The field-based sensing system includes 9 bi-polar linear Hall-effect sensors (A1301, Allegro, Worcester, MA, USA, 20 kHz bandwidth, 2.5 mV/G sensitivity) and a PM (N42 grade rectangular neodymium rare earth magnet, KJ Magnetics, Pipersville, PA, USA) attached to the linear motor as shown in the schematic in Figure 1.

The linear encoder was used for calibration as well as a basis for comparison with a resolution of 1 µm. The ratiometric analog outputs of the nine hall-effect sensors which have a sensing range of ±100 mT are sampled independently using the 16-bit differential channels on the ADC module (0.153 mV resolution) and the quadrature outputs of the optical encoder is decoded by the servo drive to translational position and transmitted to the embedded controller via CAN bus. The CAN bus also provides the communication platform for the embedded controller to transmit the desired control signal. As in Figure 1, each sensor is installed with its edges in contact with adjacent sensors and its sensing axis parallel to the magnetization of the PM. The separation distance *h* between the sensors and PM is adjusted using a precise linear rail. One complete rotation of the ball screw increases or decreases *h* by 2.54 mm (0.1″). A summary of the parameters of the sensor network and PM is consolidated in Table 3. The total closed-loop control execution time including acquisition and conversion from the analog sensors, communication via CAN Bus for encoder data and control output to linear motor, and real-time data processing (PCA and ANN online computation) is 5 ms (200 Hz).

**Figure 12:**
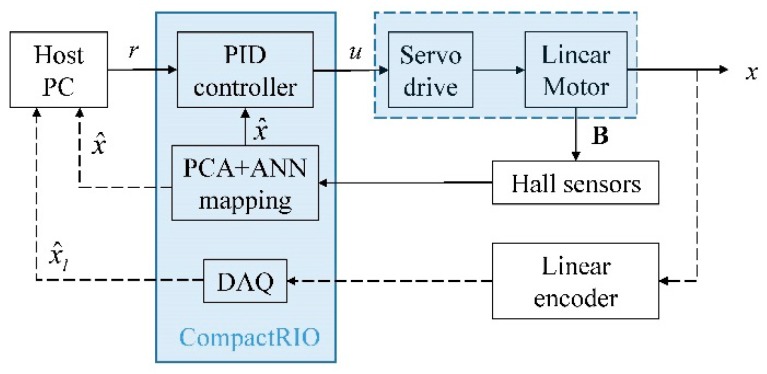
Control scheme of the linear motor using hall sensors and linear encoder.

The block diagram of the experimental setup is shown in Figure 12. The host PC specifies the reference trajectory *r* to the embedded controller (CompactRIO) and also measures and logs the instantaneous positional estimates of the field-based sensing system and linear encoder. The embedded controller computes the corresponding control signal *u* from feedback of the hall sensors or linear encoder. The servo drive amplifies the control signal transmitted to actuate the linear motor. Motion of the linear motor is detected by the change in **B** by the Hall sensors as well as directly from the quadrature output of the optical encoder.

**Figure 13:**
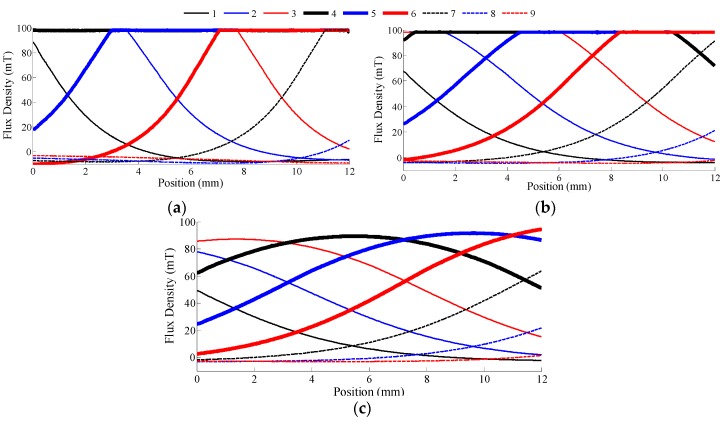
Composite **B** measurements from a nine sensor network at various separation distances. (**a**) *h*_1_ = 1.905 mm (3/4 rotations), (**b**) *h*_2_ = 3.81 mm (3/2 rotations) and (**c**) *h*_3_ = 5.715 mm (9/4 rotations).

By actuating the linear actuator in increments of 10 μm using the feedback signal of the encoder, the measured magnetic flux density (MFD) of all nine sensors at each incremental position (*N* = 1200) of the attached PM are recorded. These measurements are shown in Figure 13 at three different *h* values (*h*_1_, *h*_2_ and *h*_3_). Taking the sensor noise into consideration, a modified signal-to-noise ratio (SNR) defined by Equation (11) is used to characterize the variability in the measured signal:

(11) $$\text{SNR(dB)}=20\log_{10}\left(\sigma /Q\right)$$

where *σ* is the standard deviation of the measurements for one sensing channel; and *Q* is the standard deviation of the sensor noise. Typically, a SNR of at least 28 dB (the ratio of *σ*/*Q* is 25) is preferred to minimize noise corruption during ANN mapping. Using Equation (11), the SNR for measurements of each sensor are tabulated in Table 4. Sensors will low SNR are highlighted.

#### 3.2.1. PCA-ANN Field-Based Localization

**Figure 14:**
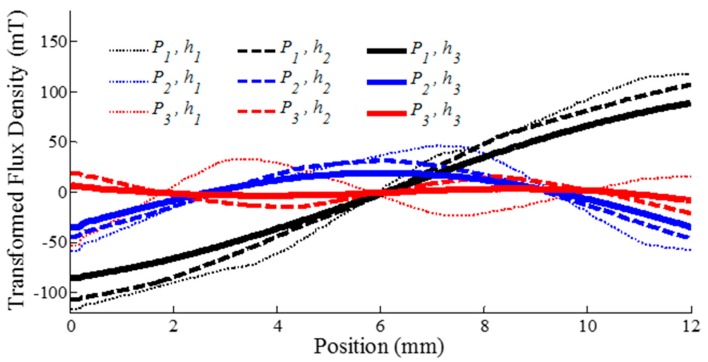
First three principal components as a function of position and separation distance.

Applying PCA on each case in Figure 13, the first three principal components are graphically illustrated as a function of PM position and three separation distances in Figure 14. The relative importance of each principal component (eigenvalue) are computed as a percentage and shown along with its corresponding SNR in Table 5.

**Figure 15:**
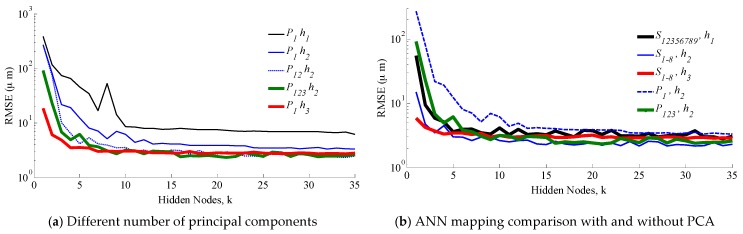

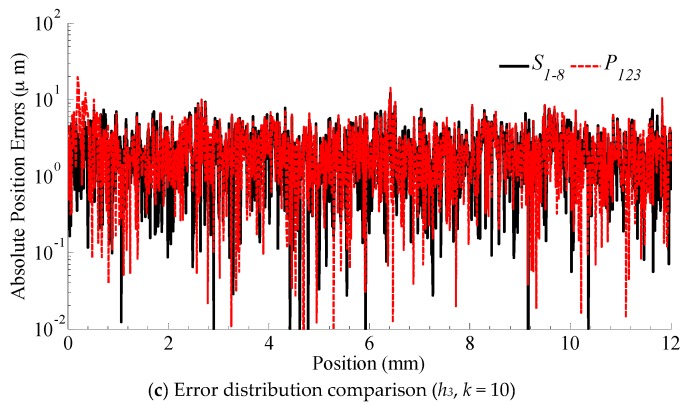
Error analysis of ANN mapping performance.

With the PCA transformed measurements, ANN models of varying hidden nodes *k* are used to characterize the measurements-position mapping. For ANN mapping, 80% of the sets will be used for training, 15% for validation and 5% for testing. The effects due to selection of ANN inputs on the mapping accuracy are presented in Figure 15, which graph the RMSE against *k*, used in the single-layer ANN mapping and absolute mapping errors against position. Figure 15a compares the effects of using only the first principal component across *h* and different number of principal components and Figure 15b,c evaluates between using the sensor and PCA filtered measurements for mapping. For mapping utilizing directly the sensor measurements, only sensor channels with at least 28 dB are considered as a viable mapping input.

#### 3.2.2. Closed-Loop Tracking Performance

**Figure 16:**
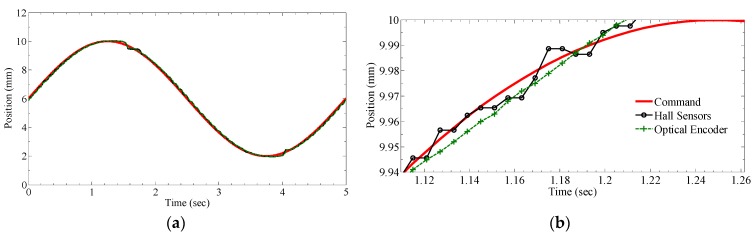
Feedback response comparison between field-based sensing and optical encoder. (**a**) Tracking performance; (**b**) Close-up view of the response at the peak in (**a**).

**Figure 17:**
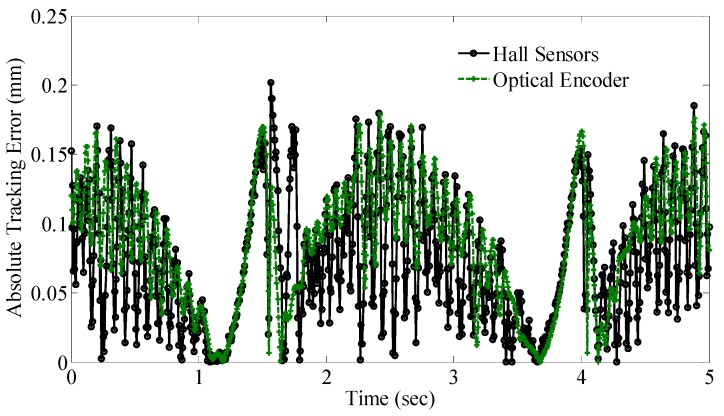
Closed-loop absolute tracking error using both sensing systems.

With a sinusoidal reference signal (0.2 Hz frequency, 4 mm amplitude centering at 6 mm), the corresponding positional estimate provided by the field-based sensing system (using a single layer, 10 hidden nodes ANN with first three principal components as inputs) and optical encoder under the same PID control for one period cycle is shown in Figure 16. A zoomed-in view of the response at the peak is also provided for clarity. The absolute tracking error between the reference command and each sensing systems is spatially compared in Figure 17. The average tracking error for the optical encoder and proposed magnetic sensing system are 0.085 mm and 0.074 mm, respectively.

## 4. Discussion

### 4.1. Numerical Results from Singular Sensor Geometrical Considerations

All normalized MFD plots (in Figure 6a) resemble a Ricker wavelet (or Mexican hat wavelet) which is characterized by its curve width and peak MFD value at zero *X*. The comparison suggests that *β* has a predominant bearing on the peak value while the effect of γ on the curve width is more pronounced. Reducing *β* resulted in a stronger field and hence a higher peak value of the wavelet due to its closer proximity to the magnetic source. Decreasing *γ* in contrast shortens the curve width, resulting in a steeper MFD plot, and it is most easily seen by comparing the MFD curves when *β* = 0.5 in Figure 6a–c.

The increase in peak value of the MFD wavelets brought by reduction in *β* is clearly reflected by the higher average AFS on the right column of Figure 6. Moreover, these curves indicate that reducing *γ* has the effect of increasing AFS for small X while decreasing AFS for large *X*. This is especially apparent when *β* and *γ* are less than unity where the AFS significantly deteriorates at *X* > 2.5.

Simultaneously reducing *β* and *γ* has an augmenting effect of increasing both the average measured MFD and AFS, but at the expense of reducing the effective range of the sensing system. This represents a design trade-off between SNR/sensitivity and range, and provides a rational basis for a multi-sensor configuration in Figure 1, which aims at maximizing SNR and sensitivity without compromising the effective range of the system.

### 4.2. Numerical Results from Dual & Multi-Sensor PCA ANN Mapping Analysis

While the MFD curves for each sensor pair (*S*_1_ and *S*_2_) in Figure 7a are symmetrical about *X* = 0, at least one of the corresponding principal components (*P*_1_ and *P*_2_) are asymmetrical. In fact, the first principal component when *δ*_21_ = 4 is a strictly increasing monotonic function. While the other sensor spacing configuration require both principal components to uniquely relate position, employing only the monotonic principal component allows the use of a single input ANN (as opposed to a two input ANN) for mapping as presented in Figure 8 albeit possessing a higher mapping error.

Of the three configurations in Table 1, despite having significantly different MFD distribution in Figure 7a, the resultant RMSE using both principal components were comparable and only separated by less than a factor of 10. The lowest RMSE was obtained when *δ*_21_ = 4 with a value of 7.53 × 10^−6^. The spatial plot in Figure 8 demonstrates a relatively consistent position error when using both *P*_1_ and *P*_2_ but noticeably higher at the ends when only *P*_1_ is employed.

Increasing the number of sensors in PCA improves sensitivity as the range of the transformed MFD being used for ANN mapping is extended. The MFD plots in of individual sensors in Figure 7a vary from −0.002 to 0.067 for a total range of 0.069. In dual sensor configuration, the corresponding range of the first principal component in Figure 7b for sensor spacings of 1, 2 and 4 are 0.083, 0.095 and 0.097, respectively. For the all-inclusive six sensor configuration, the range of the first principal component in Figure 9 is even higher, at 0.134.

As depicted in Figure 10a, the mapping performance with and without PCA is indistinguishable in absence of noise. It is noted that only three inputs were needed for the PCA- assisted mapping to achieve the same accuracy. When the sensors are corrupted with Gaussian noise, the ANN that uses PCA inputs is more resilient as visually illustrated in Figure 10b. From Table 2, at both 1% and 10% noise corruption, the RMSE of the ANN using three PCA inputs is an order of magnitude lower than a six input ANN without using PCA transformed measurements.

### 4.3. Experimental Results from PCA ANN Field-Based Localization

At small separation distances (or close proximity between the sensors and PM), many of the sensors are fully saturated (around 100 mT). In fact at *h*_1_, *S*_4_ remains fully saturated throughout the motion range. This is reflected in the low SNR in Table 4 of 7.0. As *h* is increased, saturation effects and low SNR become less of an issue. At *h*_2_ and *h*_3_, only *S*_9_ has SNR less than 28 dB. However, increasing *h* further is undesirable as it will result in reduced SNR across all sensors.

As expected, relying on a single sensor is insufficient to associate and extract position from MFD measurements due to lack of bijection. As seen in Figure 13a,b, it is exacerbated with saturation effects where the MFD measurements remains constant for multiple positions. With PCA, the 1st principal component *P*_1_ is clearly bijective for all three values of *h* as reflected in Figure 14. Hence, for any *h* value, the 1st principle component is sufficient to uniquely infer position.

The first three principal components represent an overwhelming majority of the total variability. They account for 99.47%, 99.88% and 99.97% for different *h* values respectively. In fact, the 1st principal component alone was responsible for at least 80% (For *h*_3_, it was over 90%). In addition, the SNR for the 1st principal component for all *h* (over 60 dB) was noticeably higher than any of the individual sensors SNR in Table 5. At 60 dB, σ/Q is 1000.

Using only the first principal component *P*_1_ (single input ANN model), an RMSE of 10 µm or less is attained for all values of *h*. With the exception for *h*_1_ (possibly due to the high degree of saturation across the sensors), this was achieved with only five hidden nodes. The results suggest that with PCA, the mapping performance is relatively insensitive to *h*, but related to the variability of the corresponding principle component. The larger the variability, the smaller the mapping errors.

Employing additional principal components reduces the RMSE further. At *h*_2_, using the first two and all three principal components lowers the RMSE to 2.5 µm and 2.4 µm, respectively, at *k* = 35. This modest improvement reiterates the significance of the first principal component in mapping accuracy.

While attaining sub-micron accuracy is possible using the measurements without PCA as depicted in Figure 15b, this is achieved at a cost of using higher order ANN models (6, 8 and 8-input models for *h*_1_, *h*_2_ and *h*_3_ respectively) which require larger computational overheads and memory. Moreover, the RMSE of using with and without PCA filtered measurements are comparatively insignificant. Even with hidden nodes as low as *k* = 10, the absolute error distribution in Figure 15c demonstrates that the mapping accuracy at *h*_3_ using only three principle components (*P*_1,2,3_) as ANN inputs is comparative to that using eight sensor measurements (*S*_1–8_) throughout the range of motion.

### 4.4. Experimental Results from Closed-Loop Tracking Performance

The preceding results in Figure 16 and Figure 17 show that while the proposed PCA mapping approach could reduce the computational expensiveness of the field-based method for real-time tracking, the corresponding system response is able to offer comparative performance to the system response using high resolution encoder of 1 µm. Taking account of the prices of the commercialized hall sensors and optical encoder used in the setup, the proposed PCA mapping approach provides a more cost-effective solution without loss of functionality (non-contact sensing) or precision.

## 5. Conclusions

Using a network of magnetic sensors, the position of a moving PM can be extracted with high precision via ANN mapping from concurrent measurements of all sensors. With PCA operating as a pseudo-filter, the number of ANN inputs required can be significantly reduced with minimal effect on overall accuracy while increasing computational speed; thereby improving real-time tracking control performance. Moreover, through simulations and experimental investigation, it was found that PCA has the beneficial effects of rendering sensing performance insensitive to separation distance as well as capable of coping with sensor saturation effects and noise corruption in practical applications. With a spatial network of nine magnetic sensors used in tandem with a 3-input PCA optimized ANN, positional micron accuracy of a linear motor was achieved which offered comparative performance of an optical encoder with resolution of 1 µm. As PCA transformed outputs as well as ANNs are achieved using simple algebraic operations, even in very large scale sensor networks, they can be realized easily in real-time with rudimentary analog summing circuits.

## Figures and Tables

**Table 1:** Comparison among dual-sensor configurations.

Sensor Spacing	PCA						ANN RMSE (Normalized)
	% Variability		Coefficients				
*δ*_21_	*P*_1_	*P*_2_	*C*_11_	*C*_12_	*C*_21_	*C*_22_	
1	52.2	47.8	1/√2	−1/√2	1/√2	1/√2	1.60 × 10^−5^ (*P*_1,_*P*_2_)
2	96.0	4.0	1/√2	1/√2	−1/√2	1/√2	9.00 × 10^−6^ (*P*_1,_*P*_2_)
4	78.5	21.5	1/√2	−1/√2	−1/√2	−1/√2	7.53 × 10^−6^ (*P*_1,_*P*_2_)
							1.13 × 10^−3^ (*P*_1_ only)

**Table 2:** PCA parameters and noise attenuation performance of a 6 sensor configuration.

	PCA % Variability						ANN Position Mapping		
	*P*_1_	*P*_2_	*P*_3_	*P*_4_	*P*_5_	*P*_6_	Noise	Inputs	ANN RMSE (Normalized)
	71.6	24.0	4.1	0.31	0.040	0.011	0%	*P*_123_	2.85 × 10^−6^
	**PCA Coefficient Matrix**							*S*_1–6_	2.40 × 10^−6^
*C_ij_*	*i *= 1	2	3	4	5	6	1%	*P*_123_	1.09 × 10^−2^
*j* = 1	0.309	−0.577	0.332	−0.002	0.541	−0.408		*S*_1–6_	3.13 × 10^−3^
2	−0.309	−0.577	−0.332	−0.002	−0.541	−0.408	10%	*P*_123_	1.05 × 10^−1^
3	0.499	−0.160	0.246	−0.649	−0.436	0.230		*S*_1–6_	3.32 × 10^−2^
4	−0.499	−0.160	−0.246	−0.649	0.436	0.230			
5	0.394	0.376	−0.573	−0.280	0.127	−0.529			
6	−0.394	0.376	0.573	−0.280	−0.127	−0.529			

**Table 3:** Experimental setup parameters.

Field Sensor Network			PM (Grade N42)			
*n*	*d_1_*(mm)	*d_i_*_+1_ − d*_i_* (mm)	*2w *(mm)	*l *(mm)	*c *(mm)	*M_o_* (A/m)
9	−7.27	4.09	12.7	6.35	4.76	4.67 × 10^5^

**Table 4:** Distribution of hall sensor SNR.

*h* (mm)	SNR (dB)								
	*S*_1_	*S*_2_	*S*_3_	*S*_4_	*S*_5_	*S*_6_	*S*_7_	*S*_8_	*S*_9_
1.905	54.1	59.1	56.5	7.0	53.4	59.4	57.2	37.3	32.0
3.81	52.1	57.6	55.6	40.3	53.1	58.0	55.1	42.2	21.6
5.715	49.6	54.0	53.9	46.4	52.9	55.9	51.9	42.6	27.0

**Table 5:** Relative dominance and SNR of each principal component.

*h* (mm)	% Variability, (SNR, dB)								
	*P*_1_	*P*_2_	*P*_3_	*P*_4_	*P*_5_	*P*_6_	*P*_7_	*P*_8_	*P*_9_
1.905	79.55	14.54	5.49	0.22	0.12	0.04	0.018	~0	~0
	(64.0)	(56.6)	(52.4)	(38.4)	(35.9)	(31.8)	(27.6)	(5.9)	(4.8)
3.81	86.81	10.67	1.98	0.37	0.11	0.04	0.004	~0	~0
	(63.0)	(53.9)	(46.6)	(39.3)	(34.1)	(29.9)	(19.1)	(10.7)	(−0.5)
5.715	91.50	8.20	0.27	0.03	0.001	0.0002	0.0001	~0	~0
	(61.0)	(50.6)	(35.8)	(26.3)	(12.8)	(4.4)	(1.2)	(−0.2)	(−1.4)
